# Synergistic Interaction of Chloroquine with *Artemisia kopetdaghensis* Semipolar Extract Against *Plasmodium berghei*: Histopathological and Immunological Studies in a Mouse Model

**DOI:** 10.5812/ijpr-147234

**Published:** 2024-09-24

**Authors:** Roya Amirian, Mustafa Ghanadian, Hamed Fouladseresht, Azar Baradaran, Seyed Mohammad Abtahi, Bahareh Basirpour, Maryam Fattahian, Seyed Mahmoud Mousavi, Parastoo Hassani-Abharian, Hajar Shabandoust, Seyedamirmehdi Hejazi Dehaghani, Seyed Hossein Hejazi

**Affiliations:** 1 Department of Parasitology and Mycology, School of Medicine, Isfahan University of Medical Sciences, Isfahan, Iran; 2 Department of Pharmacognosy, Isfahan Pharmaceutical Sciences Research Center, School of Pharmacy, Isfahan University of Medical Sciences, Isfahan, Iran; 3 Department of Immunology, School of Medicine, Isfahan University of Medical Sciences, Isfahan, Iran; 4 Infectious Diseases and Tropical Medicine Research Center, Isfahan University of Medical Sciences, Isfahan, Iran; 5 Department of Pathology, Isfahan University of Medical Sciences, Isfahan, Iran; 6 Department of Parasitology, School of Medicine, Mazandaran University of Medical Sciences, Sari, Iran; 7 School of Pharmacy and Pharmaceutical Sciences, Isfahan University of Medical Sciences, Isfahan, Iran; 8 Department of Parasitology and Mycology, Skin Diseases and Leishmaniasis Research Center, School of Medicine, Isfahan University of Medical Sciences, Isfahan, Iran; 9 School of Medicine, Padova University, Padova, Italy

**Keywords:** Malaria, Synergism, Cytokine, * Artemisia kopetdaghensis*, * Plasmodium berghei*

## Abstract

**Background:**

Malaria parasites have gradually developed resistance to commonly used antimalarial drugs. For decades, chloroquine was the most widely used drug to eradicate malaria. However, with the spread of chloroquine resistance, many countries have adopted combination therapies that utilize two drugs acting synergistically instead of monotherapy. In this study, the synergistic effect of chloroquine and the semipolar extract of *Artemisia kopetdaghensis*. Semipolar extract (SPE) was investigated in vivo through pathological and parasitological studies on mouse model.

**Methods:**

Sixty female Balb/c mice infected with the *Plasmodium berghei* (*P. berghei*) parasite were treated with different concentrations of the semipolar extract of *Artemisia kopetdaghensis* (SPE) according to the protocol. The mean percentage of parasitemia, the mean survival time of the mice, the serum levels of IFN-γ, IL-4, IL-17, and TGF-β, and the effects of the SPE on the kidney, spleen, and liver tissues were investigated and compared across different treatment groups. The data were analyzed using Bonferroni, ANOVA, and Tukey tests.

**Results:**

The semipolar extract of *Artemisia kopetdaghensis* (SPE) demonstrated better therapeutic effects in both synergistic and monotherapy conditions compared to chloroquine alone. The combination of chloroquine and SPE resulted in the lowest parasitemia rate, the highest percentage of parasite inhibition, and the longest average survival time. Pathological studies showed no signs of acute toxicity in the organs.

**Conclusions:**

This study demonstrated that using chloroquine in combination with *Artemisia kopetdaghensis* semipolar extract has synergistic effects in reducing parasitemia, enhancing the inhibitory effect on parasite growth and reproduction, and balancing the host immune system.

## 1. Background

Malaria, a significant global health threat, poses a continuous danger to those who travel to tropical endemic areas. This disease is a hemato-protozoan parasitic infection transmitted through the bites of infected female *Anopheles* mosquitoes. If not identified and treated promptly, this mosquito-borne infection can result in a fatal condition. One of the greatest obstacles to controlling malaria has been the spread of resistance to all classes of antimalarial drugs, contributing to the recent increase in malaria-related mortality, particularly in Africa. Prolonged efforts are urgently needed to combat drug resistance. Until recently, chloroquine had been the most widely used drug to eradicate malaria throughout the last century. However, its extensive use over the decades has led to chloroquine resistance in the parasite species responsible for most human malaria cases. With the spread of chloroquine resistance, many countries have adopted combination therapies composed of two drugs acting synergistically instead of monotherapy. The simultaneous use of two or more drugs aims to harness the synergistic or additive potential of the drugs to improve therapeutic efficacy and decrease the risk of resistance to each of the individual components. 

*Artemisia kopetdaghensis* is a notable aromatic plant characterized by the presence of a broad range of sesquiterpene lactones (STLs) and methoxylated flavones, which have been previously identified in its semipolar extract (1, 2). It has been well established that STLs and flavones exhibit remarkable biological effects, including antimalarial (3-5), cytotoxic (6), antibacterial (7), antifungal, and antioxidant (8) activities. These facts prompted us to undertake a biological investigation of the semipolar fraction of *A. kopetdaghensis*, containing both sesquiterpenes and flavones, in combination with chloroquine to assess their synergistic antimalarial activity against *P. berghei* in mouse model.

## 2. Objectives

In the present study, we investigated the mean percentage of parasitemia, the mean survival time of the mice, the serum levels of IFN-γ, IL-4, IL-17, and TGF-β, and the effects of the SPE on the kidney, spleen, and liver tissues of mice across different treatment groups. The data were analyzed using Bonferroni, ANOVA, and Tukey tests.

## 3. Methods

### 3.1. Plant Material and Extraction Process

Aerial parts of *Artemisia kopetdaghensis* were collected from Bojnord, North Khorasan province, northeast of Iran, in mid-September 2016. The plant was authenticated by Mohammad Reza Joharchi at the Department of Botany, Herbaceous Sciences Research Centre, Ferdowsi University of Mashhad, Iran. A voucher specimen (SAM-4005 of the Herbarium) has been preserved at the School of Pharmacy and Pharmaceutical Sciences, Isfahan University of Medical Sciences, Isfahan, Iran. In a previously conducted study, 9 kg of the dried plant was powdered and macerated in a mixture of chloroform and acetone (CH2Cl2:Me2CO, 2:1, 40 L × 3) at room temperature. The extract was subsequently concentrated using a rotary evaporator at 40ºC under reduced pressure (2).

### 3.2. Standardization of the Extract by Total Flavonoid Content

Both methoxylated flavones and sesquiterpene lactones are responsible for the observed effects. Therefore, the total flavonoid content (TFC) of the semipolar extract was analyzed using the complexation method, with some modifications (1-9). For this, 100 mg of the sample was mixed with 100 μL of a saturated solution of aluminum chloride in methanol. Two drops of acetic acid were added, and the volume was adjusted to 1 mL with methanol. The mixture was then incubated for 60 minutes at room temperature. The absorbance was measured at a wavelength of 415 nm against a blank sample, and the results were reported as mean ± standard deviation (SD).

### 3.3. Phytochemical Analysis

Briefly, the semipolar extract was filtered through a C-18 filled sintered glass funnel using a methanol: Water (70: 30) mixture and then chromatographed on a silica gel column. The column was washed with a gradient of hexanes: Ethyl acetate (97: 3 to 30: 70; Fr.1 – Fr.14). Fractions containing sesquiterpene lactone signals, as identified by ^1^H-NMR experiments, were selected for further purification. These fractions were purified by recrystallization or high-performance liquid chromatography (HPLC) on a YMC silica column using a chloroform: Methanol (90: 10) solvent system to yield pure sesquiterpene lactone (SL) compounds, as previously reported elsewhere (2).

### 3.4. In Vivo Antimalarial Tests

#### 3.4.1. Animals and Parasites

Balb/c healthy primiparous non-pregnant female mice, aged 6 to 8 weeks with an average weight of 20 to 30 grams, were obtained from the Pasteur Institute, Tehran, Iran. The mice were housed in standard insulated autoclavable cages with a temperature maintained between 20 to 24ºC and a 12-hour light-dark cycle. Water and food were freely available to them. Throughout the experiment, ethical guidelines for handling and caring of laboratory animals were strictly followed according to the approved protocol by the Ethical Committee of Laboratory Animals of Isfahan University of Medical Sciences. *Plasmodium berghei* of the SPH-TUMS strain was obtained from the Department of Parasitology and Mycology, School of Medicine, Isfahan University of Medical Sciences.

#### 3.4.2. Inoculation of the Parasite

A valume of 0.2 mL of blood containing 1 × 10^7^ red blood cells infected with the parasite was injected intraperitoneally into each mouse. To check the contamination level, 0.1 mL of blood was taken from the ends of the mice tails, thin smears were prepared and after Gimsa staining, they were examind under a microscope using immersion oil and 40x objective lenses, and examined under a microscope. After 5 days, infected blood from donor mice with a 20% parasitemia level was collected by cardiac puncture after anesthesia and placed into heparinized Falcon tubes. To infect the mice in the test groups, a 0.2 mL dose containing 10^7^ infected red blood cells diluted in physiological serum was injected into each mouse.

### 3.4.3 Grouping and Dosage Regimen Designing for Animals

The antimalarial effect was evaluated in all groups of malaria-infected mice following the 5-day test treatment protocol described by Misganaw et al. (10). The infected mice were divided into 10 groups of 6 mice each. Three groups received *Artemisia kopetdaghensis* semipolar extract (SPE) at doses of 50, 100, and 150 mg/kg, administered intraperitoneally (I.P.) for 3 consecutive days starting 48 hours (day 2) after infection. The fourth group received a combination of SPE and chloroquine (100 mg/kg of SPE + 10 mg/kg of chloroquine). Dimethyl sulfoxide (DMSO) (10%) and Tween 80 (2%) in Phosphate-buffered saline (PBS) were used as the vehicle. The negative control group received the vehicle, while the positive control group received chloroquine at a dose of 10 mg/kg.

On days 2, 4 and 7 of experiment blood was collected from the tails of the mice for parasitological analysis using the standard Giemsa staining method through the following formula:


$$Parasitemia\;\left(\%\right)=\frac{Number\;of\;PRBC\;in\;5\;fields}{Number\;of\;all\;RBC}\;\times 100$$

$Parasite\;inhibition\;\left(\%\right)=\;\frac{Average\;\%\;parasitemia\;in\;negative\;control-Average\;\%\;parasitemia\;in\;treatment\;groups\;}{Average\;\%\;parasitemia\;in\;negative\;control\;}\;\times 100$

From the start of the mice infection (day 0) to the end of the study (day 29), the number of deaths in each group was recorded, and the mean survival time (MST) was calculated for each group using the following formula:


$$MST=\frac{Sum\;of\;survival\;time\;of\;all\;mice\;in\;each\;group\;(day)}{Total\;number\;of\;the\;mice\;in\;the\;group}\;\times 100$$

#### 3.4.4. Analysis of Serum Levels of Cytokines IFN-γ, IL-4, IL-17, and TGF-β

After the mice were anesthetized with ketamine-xylazine, blood was collected from the corners of their eyes on days 0, 2, 4, and 7 using a heparinized capillary tube. Two hours later, the samples were centrifuged for 15 minutes at 3000 rpm, and the serum was carefully collected without mixing with the buffy coat layer. The serum samples were stored at -20°C in cryovials until the evaluation of interferon-gamma, interleukin 4, interleukin 17, and TGF-beta cytokines using a standard ELISA kit, following the instructions provided with the test kit. ELISA was performed using R&D system kits (PBL Biomedical Laboratories, NJ, USA) (11).

#### 3.4.5. Histopathology

After the mice were anesthetized with ketamine-xylazine, the liver, spleen, and kidney tissues were separated, washed in sterile physiological serum, and placed in 10% formalin. After 24 hours, paraffin blocks were prepared from the tissue samples, sliced into 5 μm sections, and stained with hematoxylin and eosin. The changes in the liver and kidney tissue sections, along with the alterations in cell accumulation within the tissues, were examined by a histopathologist using a Nikon binocular microscope (12).

### 3.5. Statistical Analysis

The results were compared using SPSS software (version 22, IBM Corporation, Armonk, NY) with a one-way analysis of variance (One-Way ANOVA) and Tukey post hoc test. A P-value of less than 0.05 was considered statistically significant, and the data were expressed as mean ± SD.

## 4. Results

### 4.1. Sample Standardization

The extract was standardized by quantifying the TFC using the aluminum chloride (AlCl_3_) method at 425 nm. The semipolar extract (SPE) was standardized to 2.49 ± 0.16 mg equivalent quercetin per 100 g of dry weight.

The plant extract was also analyzed in the phytochemistry lab to identify the sesquiterpene lactones (STLs) previously reported (2). Briefly, it contained several eudesmanane-type STLs, including 4-epi-persianolide A, 3α,4-epoxypersianolide A, persianolide A, 1β-hydroxy-11-epi-colartin, 1β,8α-dihydroxy-11α,13-dihydrobalchanin, and 11-epi-artapshin as the main lactone (2).

### 4.2. The Average Percentage of Parasitemia in Each Treatment Group

**Figure 1.**
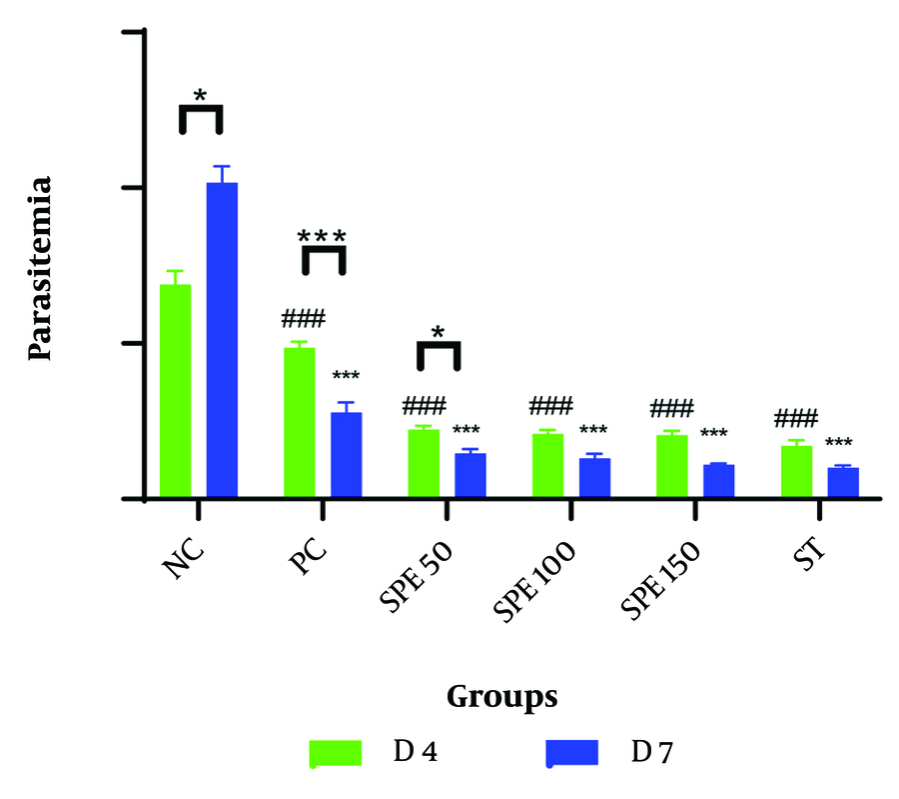
Comparison of the average percentage of parasitemia in treatment groups: Semipolar extract (SPE) at doses of 50, 100, and 150 mg/kg as well as synergism treatment (ST) of SPE (100 mg/kg) + chloroquine (10 mg/kg), vehicle as negative control (NC), and chloroquine (10 mg/kg) as positive control (PC) on days 4 and 7 after parasite injection. Treatment was started on day 2 after the parasite injection. * P < 0.05, *** P < 0.0001 within group between days 4 and 7; ### P < 0.0001 between groups on day 4; * P < 0.05, *** P < 0.0001 between groups on day 7 were significant.

The mean percentage of parasitemia was measured on days 2, 4, and 7 for each group. A comparison of the average percentage of parasitemia in the groups treated with different concentrations of SPE, as well as the synergistic group, negative control group, and positive control group on days 4 and 7 post-infection, is presented in Table 1 and Figure 1. All data followed a normal distribution, and the data analysis was performed using the One-Way ANOVA test.

**Table 1.** The Average Percentage of Parasitemia in Different Study Groups

Examination Group	Average Percentage of Parasitemia/Day After Treatment		P-Value Pairwise
**Day**	**4**	**7**
**Negative control (Tween 80 + DMSO 10%)**	6.89 ± 0.44	10.17 ± 0.52	< 0.001
**Chloroquine**	4.86 ± 0.50	2.78 ± 0.33	< 0.001
**SPE**			
50 mg/kg	2.23 ± 0.29	1.47 ± 0.36	< 0.001
100 mg/kg	2.09 ± 0.32	1.30 ± 0.40	0.04
150 mg/kg	2.06 ± 0.32	1.10 ± 0.09	< 0.001
**SPE + chloroquine (100 mg/kg + 10 mg/kg)**	1.71 ± 0.48	1.01 ± 0.17	< 0.001

Abbreviation: SPE, semipolar extract.

### 4.3. Parasite Inhibition Percentage in Treatment Groups

**Figure 2.**
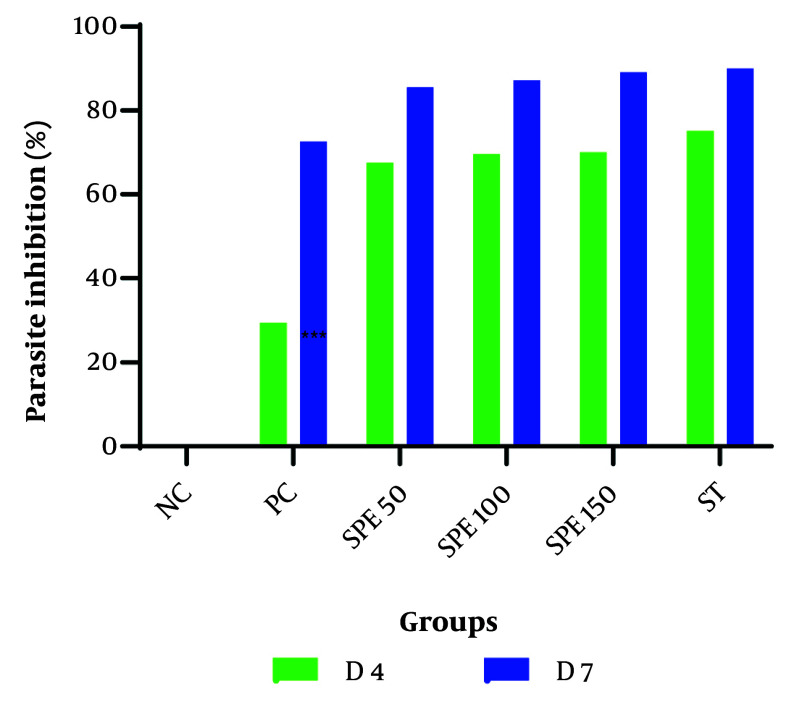
The average percentage of parasite inhibition in treatment groups: Semipolar extract (SPE) at doses of 50, 100, and 150 mg/kg as well as synergistic treatment (ST) of SPE (100 mg/kg) + chloroquine (10 mg/kg), vehicle as negative control (NC), and chloroquine (10 mg/kg) as positive control (PC) on days 4 and 7 after parasite injection.

The amount of parasite inhibition was calculated, as shown in Table 2. The percentage of parasite inhibitory activity on day 7 after treatment with different concentrations of the SPE 50, 100, and 150 mg/kg was found to be 85.54%, 87.17%, and 89.12%, respectively. In the synergistic group, this percentage reached 90.01%, indicating a higher inhibition rate than all the monotherapy groups. The average percentage of parasite inhibition for the treatment groups, positive control, and negative control is shown in Figure 2. 

**Table 2.** Percentage of Parasite Inhibition by Different Concentrations of SPE and Synergistic Group with Chloroquine, Negative Control Group, and Positive Control Group on Days 4 and 7 after Parasite Injection

Examination Groups	Parasite Inhibition Percentage (%)	
	4	7
Day		
**Negative control (Tween% 80 + DMSO 10%)**	0	0
**Chloroquine**	29.34	72.65
**SPE**		
(50 mg/kg)	67.56	85.54
(100 mg/kg)	69.59	87.17
(150 mg/kg)	70.05	89.12
**SPE (100 mg/kg) + chloroquine (10 mg/kg)**	75.13	90.01

Abbreviation: SPE, semipolar extract.

### 4.4. Mean survival time

**Figure 3.**
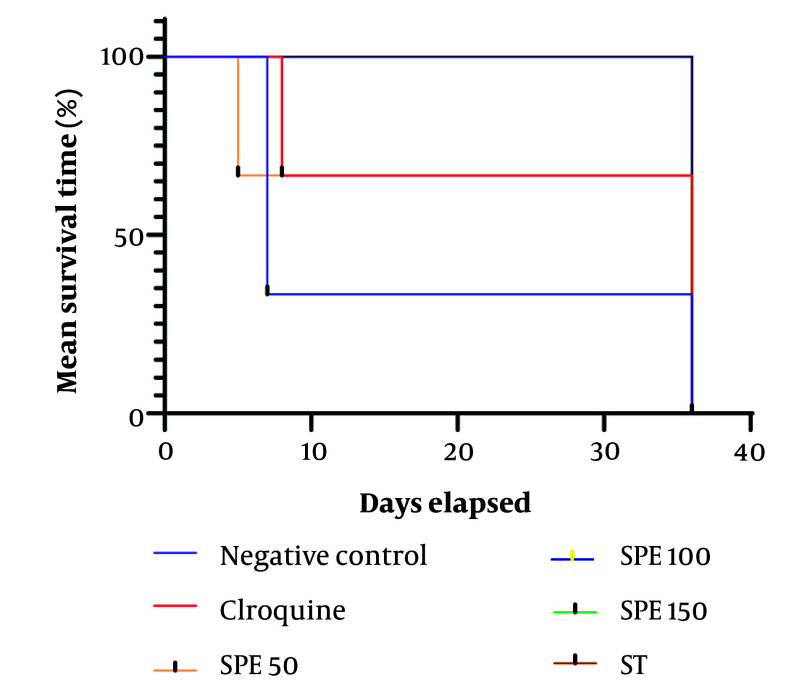
The average survival time percentage for each group

The average survival time of the mice for each group is presented in Table 3. The Kolmogorov-Smirnov test indicated that the data distribution is not normal; therefore, the Kruskal-Wallis test was used to compare the average survival time across different experimental groups. The average survival time for the positive control group, which received 10 mg/kg of chloroquine, was calculated to be 26.67 ± 14.46 days. For the negative control group, this average was 16.67 ± 14.98 days. The average survival time for the other treatment groups was 36.00 ± 00 days, except for the treatment group receiving 50 mg/kg of SPE, which had an average survival time of 16.01 ± 25.67 days. According to the Kruskal-Wallis test, these averages are significantly different from each other (P-value < 0.0001) (Figure 3). 

**Table 3.** The Average Survival Time for The Studied Groups in A 36-Day Experiment

Examination Groups	Dose (mg/kg)	Mean Survival Time (Day)
**Negative Control**	Tween 80% + DMSO 10%	16.67
**Chloroquine**	10	26.67
**SPE**		
1	50	25.67
2	100	36.00
3	150	36.00
**ST (SPE + Chloroquine)**	100 + 10	36.00

Abbreviations: SPE, semipolar extract; ST, synergistic treatment.

### 4.5. Histopathological Findings

**Figure 4.**
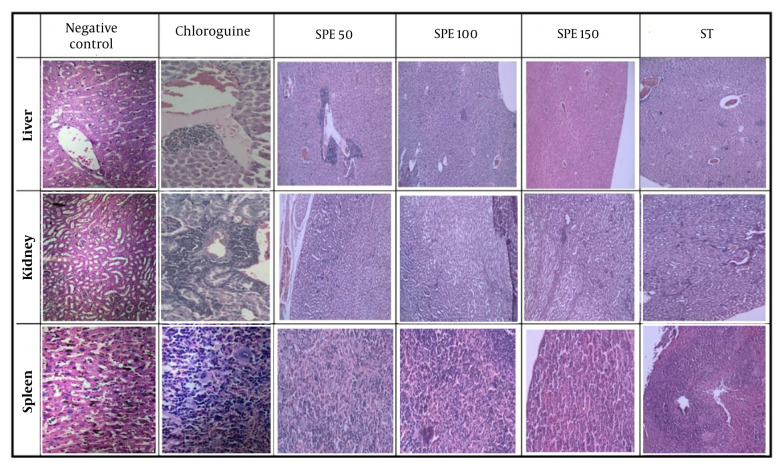
The histopathological analysis of the sections prepared from the liver, kidney, and spleen tissues of the studied groups. Semipolar extract (SPE) at doses of 50, 100, and 150 mg/kg as well as synergistic treatment (ST) of SPE (100 mg/kg) + chloroquine (10 mg/kg), vehicle as negative control, and chloroquine (10 mg/kg) as positive control on day 36.

The histopathological analysis of the sections prepared from the liver, kidney, and spleen tissues of the mice was conducted by the pathologist (Figure 4). 

In the negative control group, extramedullary hematopoiesis, abundant brown pigments, and accumulation of hemozoin were observed in the spleen tissue. In the liver, the presence of numerous pigments, expansions, and inflammations in both the portal space and the central lobular veins indicated parasite penetration.

In the positive control group, adverse drug effects were identified, including severe infiltration of inflammatory cells, along with dilation of the portal triads and central lobular veins in the liver tissue. In the kidney tissue, nephrotoxicity, tubule damage, and tubulitis were appreciable. In the spleen tissues, extramedullary hematopoiesis and macrophages containing hemosiderin were observed.

For the group receiving 50 mg/kg SPE, severe congestion, dilation of blood vessels, and severe infiltration were evident in the liver tissue. In the kidney tissue, perinephric fat and severe infiltration were recognized. Numerous hemosiderin-laden macrophages were seen in the extramedullary spleen, along with megakaryocytes. The sinusoids were dilated, and the tissue damage was very severe.

In the mice receiving 100 mg/kg of SPE, only a few infiltrations were observed in the liver tissues. There were no signs of necrosis, thrombosis, or bleeding, the hepatocytes appeared healthy, and no disintegration was detected, indicating that this concentration is not toxic to liver cells. Additionally, the kidney tissue was healthy, and only partial megakaryocytes and extramedullary hematopoiesis were diagnosed in the spleen.

For the group receiving 150 mg/kg of SPE, slight infiltration was observed in the liver tissue. There were no signs of bleeding, thrombosis, or necrosis, but partial hematopoiesis and a few megakaryocytes were detected in the extramedullary spleen.

In the synergistic group, the kidney tissue appeared relatively healthy, with only mild lymphocytic infiltration and partial congestion. In the liver tissue, minor infiltration was observed around the hepatocytes. The spleen was in the best condition, with negligible extramedullary hematopoiesis.

### 4.6. The Serum Levels of Cytokines

**Figure 5.**
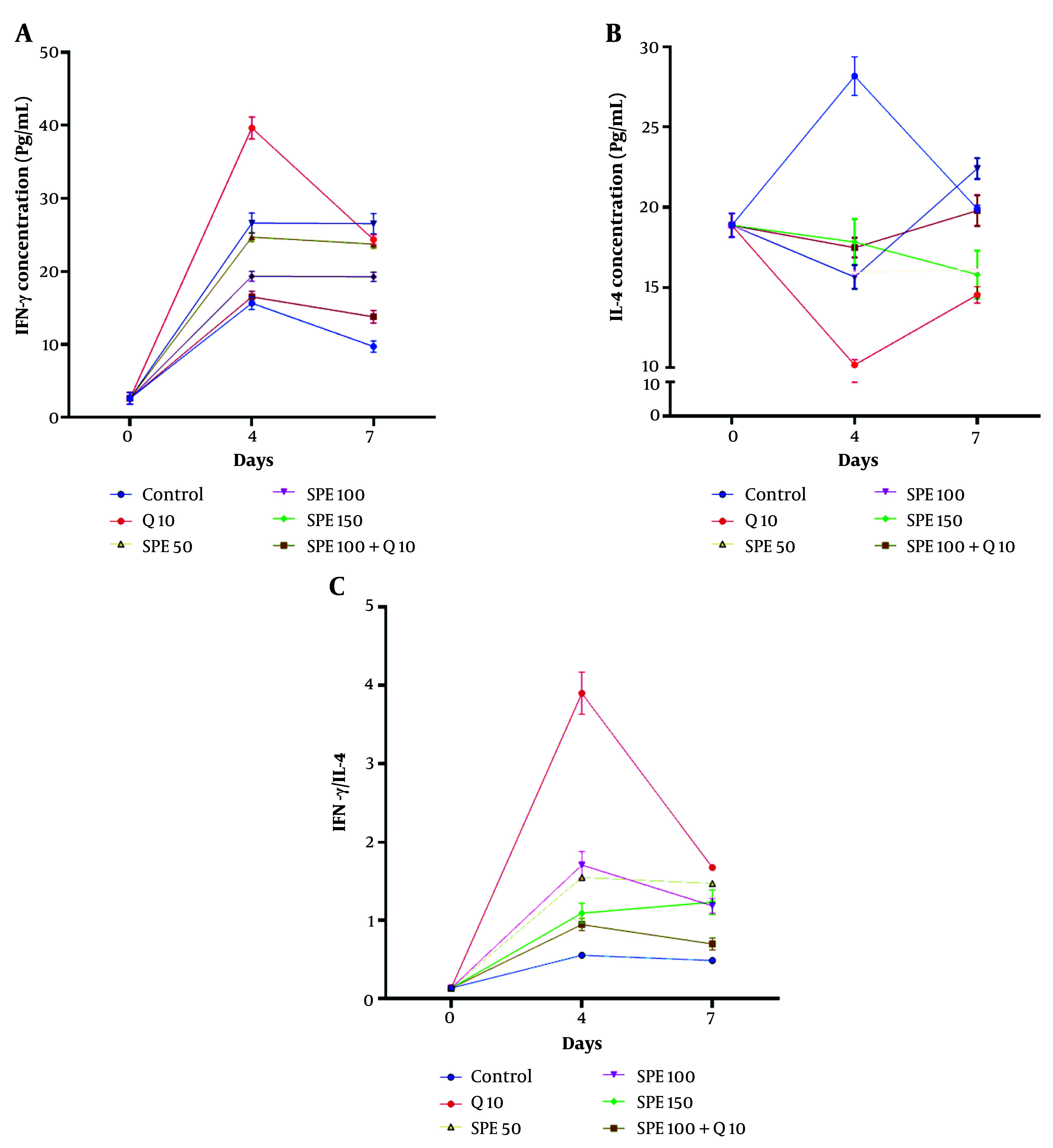
Changes in IFN-γ (A), IL-4 (B), and IFN-γ/ IL-4 ratio (C) of serum levels on days 0, 4, and 7 in groups exposed to different treatments. Semipolar extract (SPE) at doses of 50, 100, and 150 mg/kg (SPE50, SPE100, SPE150) as well as synergistic treatment (ST) of SPE (100 mg/kg) + chloroquine (10 mg/kg), vehicle as negative control, and chloroquine (10 mg/kg) as positive control.

Figure 5A shows the changes in serum levels of IFN-γ on days 0, 4, and 7 in mice treated with different concentrations of chloroquine (Q) and SPE (single or synergistic). The Kruskal-Wallis test revealed a significant difference in IFN-γ serum levels between treatments on both day 4 and day 7 (Kruskal-Wallis test, P < 0.0001). On day 4, the groups treated with the combination of Q10 (chloroquine, 10 mg/kg) and SPE100 (semipolar extract, 100 mg/kg) showed the highest serum levels of IFN-γ compared to the control group (Multiple comparison test, P = 0.0002 and P = 0.0158, respectively). On day 7, the highest serum level of IFN-γ compared to the control group was observed in the group treated with SPE100 (Multiple comparison test, P = 0.0026).

Figure 5B depicts the changes in IL-4 serum levels on days 0, 4, and 7 in mice treated with different concentrations of Q and SPE (single or synergistic). The Kruskal-Wallis test showed a significant difference in IL-4 levels between treatments on both day 4 and day 7 (Kruskal-Wallis test, P < 0.0001). On day 4, the Q10, SPE100, and SPE50 groups exhibited the lowest serum levels of IL-4 compared to the control group (Multiple comparison test, P < 0.0001, P = 0.0276, and P = 0.0473, respectively). However, on day 7, only the Q10 group showed a significantly lower IL-4 serum level compared to the control group (Multiple comparison test, P = 0.0158).

Figure 5C illustrates the IFN-γ/IL-4 serum levels on days 0, 4, and 7 in mice treated with different concentrations of Q and SPE (single or synergistic). The Kruskal-Wallis test indicated a significant difference in the IFN-γ/IL-4 serum level ratio between treatments on days 4 and 7 (Kruskal-Wallis test, P < 0.0001). A comparison between groups on day 4 revealed that the groups treated with Q10, SPE50, SPE100, and SPE150 had the highest IFN-γ/IL-4 serum level ratios compared to the control group (Multiple comparison test, P < 0.0001, P = 0.0002, P = 0.0026, and P = 0.0158, respectively). Similarly, on day 7, the groups treated with Q10, SPE50, SPE100, and SPE150 exhibited the highest IFN-γ/IL-4 serum ratios compared to the control group (Multiple comparison test, P = 0.0014, P < 0.0001, P < 0.0001, and P = 0.0158, respectively).

**Figure 6.**
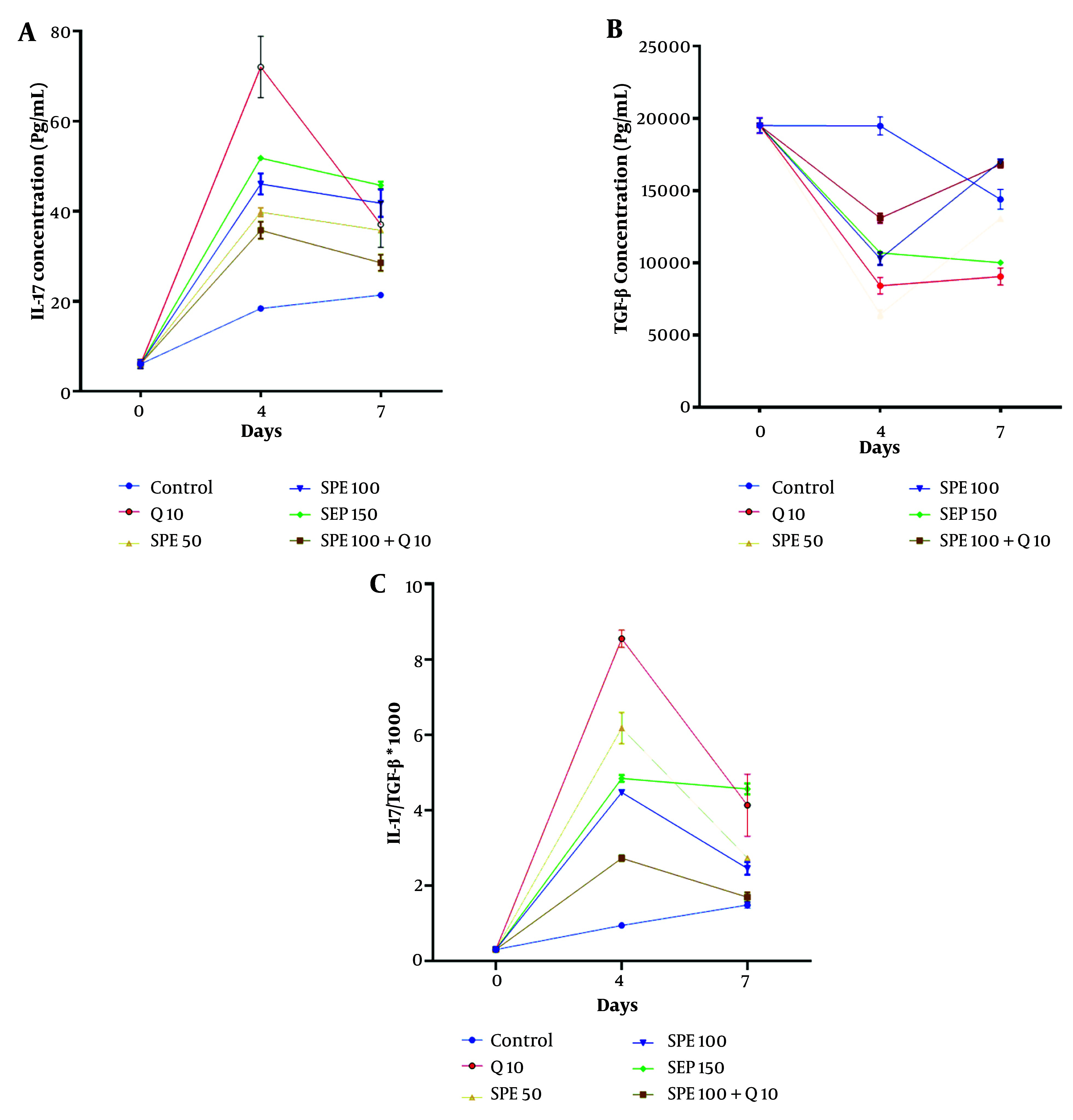
Changes in IL-17 (A), TGF-β (B), and IL-17 /TGF-β ratio (C) of serum levels on days 0, 4, and 7 in groups exposed to different treatments. Semipolar extract at doses of 50, 100, and 150 mg/kg [semipolar extract (SPE)50, SPE100, SPE150] as well as synergistic treatment (ST) of SPE (100 mg/kg) + chloroquine (10 mg/kg), vehicle as negative control, and chloroquine (10 mg/kg) as positive control.

Figure 6A depicts the alterations in IL-17 serum levels on days 0, 4, and 7 in mice treated with different concentrations of chloroquine (Q) and SPE (single or synergistic). The Kruskal-Wallis test indicated a significant difference in IL-17 levels between treatments on days 4 and 7 (Kruskal-Wallis test, P < 0.0001). On day 4, the groups treated with Q10, SPE150, SPE100, and SPE50 had the highest serum levels of IL-17 compared to the control group (Multiple comparison test, P < 0.0001, P < 0.0001, P = 0.0014, and P = 0.0026, respectively). Conversely, on day 7, the groups treated with SPE150 and SPE100 showed the highest IL-17 serum levels compared to the control group (Multiple comparison test, P = 0.0004 and P = 0.0276, respectively).

Figure 6B illustrates the changes in TGF-β serum levels on days 0, 4, and 7 in mice treated with different concentrations of chloroquine (Q) and SPE (single or synergistic). The Kruskal-Wallis test revealed a significant difference in TGF-β levels between treatments on both day 4 and day 7 (Kruskal-Wallis test, P < 0.0001). On day 4, the SPE50 and Q10 groups had the lowest TGF-β serum levels compared to the control group (Multiple comparison test, P < 0.0001 and P < 0.0001, respectively). On day 7, the Q10 group had the lowest TGF-β serum level (Multiple comparison test, P = 0.0473).

Figure 6C shows the changes in the IL-17/TGF-β serum level ratio on days 0, 4, and 7 in mice treated with different concentrations of chloroquine (Q) and SPE (single or synergistic). The Kruskal-Wallis test showed a significant difference in the IL-17/TGF-β serum level ratio between treatments on days 4 and 7 (Kruskal-Wallis test, P < 0.0001). On day 4, the Q10, SPE50, SPE150, and SPE100 groups exhibited the highest IL-17/TGF-β serum ratios compared to the control group (Multiple comparison test, P < 0.0001, P < 0.0001, P = 0.0014, and P = 0.0158, respectively). Conversely, on day 7, the SPE150, Q10, SPE50, SPE100, and ST groups displayed the highest IL-17/TGF-β serum ratios compared to the control group (Multiple comparison test, P = 0.0473, P = 0.0014, P = 0.0158, P < 0.0001, and P = 0.0004, respectively).

## 5. Discussion

The antimalarial activities of the semipolar extract of *A. kopetdaghensis* were investigated individually and in combination with chloroquine against *P. berghei* infection in mice over a five-day test period, using parasitological, histopathological, and immunological factors. 

In the study, the semipolar extract, both in combination and as a single agent, showed superior therapeutic effects in terms of parasitemia rate, percentage of parasite inhibition, histopathology, IFN-γ/IL-4, and IL-17/TGF-β balance compared to chloroquine alone. The group treated with a combination of chloroquine and semipolar extract demonstrated the lowest parasitemia rate, the highest percentage of parasite inhibition, and the longest average survival time. 

The average survival time of the mice in the treated groups, especially those in the synergistic group, was dose-dependently longer than that of the negative control group, confirming a substantial therapeutic effect. 

In groups treated with the semipolar extract, parasitemia on day 7 decreased in a dose-dependent manner, particularly when used in combination with chloroquine. The extent of parasite inhibition suggests effective parasite control. Immunological investigations conducted in this study demonstrate that, in addition to its effects on the parasite, the extract can also improve health outcomes by enhancing the host immune response; the best cytokine modulation effects were observed in the group treated with both chloroquine and the semipolar extract. In a study by Sakaivan Onjaijan and somsak vongsavath., combined treatment of mice infected with *P. berghei* using allicin and artesunate showed consistent results with the present study. Similarly, allicin significantly reduced parasitemia in a dose-dependent manner, enhanced the host immune response, and helped the treated mice survive longer (13). Ochora et al. studied the antiplasmodial activities of artemether and lumefantrine in combination with Securidaca long pedunculate Fresen (Polygalaceae) and identified a strong synergistic interaction between the two drugs, reducing the emergence of drug-resistant strains and decreasing the amount of drug required (14).

The SPE used in this study, whether as a single treatment or in combination with chloroquine, did not cause any irreversible histopathological damage to the kidney, spleen, or liver tissues of the studied animals. These results are consistent with research by Forkuo et al. on the co-administration of indoloquinoline and certain artemisinin derivatives, where no necrosis, steatosis, chronic inflammatory infiltration, or degenerative changes in the treated animals were reported (15). In the current study, malaria pigments in the treated groups were significantly reduced compared to the negative control group. In the spleen sections of all groups, megakaryoblasts and megakaryocytes were considerably fewer in the synergistically treated groups than in others. Megakaryocytes, indicative of hematopoiesis in spleen tissue and compensating for anemia symptoms caused by malaria, were reduced in the mice treated synergistically, suggesting better disease control. 

Cytokine variation data in this study revealed that the synergistic treatment had substantial modulating effects compared to the control. In a similar study, David et al. investigated the impact of Diospyros mespiliformis Hochst on mice infected with *P. berghei* and demonstrated its immunomodulating effects on the mitochondria (16).

This study investigated the different patterns of IFN-γ, IL-4, IL-17 and TGF-β serum levels in mice. These patterns, associated with differences in parasite load and tissue complications, underscored the significance of cytokines at different stages of host defense against the parasite. Notably, during peak parasitemia and tissue complications under different treatments, there was an increase in IL-4 and IL-17 levels and a decrease in IFN-γ and TGF-β levels; elevated IL-4 levels and reduced levels of effective cytokines like IFN-γ potentially lead to diminished immune responses against intracellular parasites. At the same time, the increase in IL-17 levels and decrease in TGF-β levels suggest a shift from the Th1/IFN-γ axis to the Th17/IL-17 axis in immune responses due to inadequate control of the intracellular parasite reservoir. While the Th17/IL-17 axis is effective against extracellular parasites, it fails to eliminate intracellular parasites, leading to chronic inflammation, tissue damage, and organ complications. Additionally, a decrease in the Treg/TGF-β axis response was indicated by lower TGF-β levels.

These findings align with those of Angulo and Manuel., who investigated the impact of cytokines on malaria pathogenesis and protection. They demonstrated that malaria pathogenesis is a complex process in which both cellular and humoral components of the acquired immune system are crucial in eliminating Plasmodium from the infected host. The Th1 and Th2 subsets of the cellular arm play vital roles in controlling parasitemia at different stages, clearing the parasite via distinct mechanisms, preventing excessive inflammation, and promoting tissue repair. During the initial control of the parasite, the Th1 subset and its associated cytokines, particularly IFN-γ (Th1/IFN-γ axis), are of utmost importance. It is crucial to reduce Th1 responses after parasite removal by other immune cells, i.e., Th2 and its associated cytokines such as IL-4 (IL-4/Th2 axis), to prevent inflammatory tissue destruction. After the Th1 peak, when the Th1-Th2 balance is disrupted to induce anti-parasitic responses, increasing the Th2 levels is vital to counteract the inflammatory effects of Th1 and re-establish the Th1-Th2 balance (17). The present study demonstrates the critical role of cytokines in controlling and inhibiting the parasite, thus confirming the results of the study by Angulo and colleagues. IFN-γ and IL-17 levels increased simultaneously up to day 4 in both the chloroquine group (positive control) and the group with synergistic interaction of chloroquine with semipolar extract (ST group), with the latter showing the best performance among all groups in this study. Subsequently, both cytokines decreased from day 4 to day 7. These alterations, associated with the decrease in IL-4 and TGF-β cytokines during the first four days of infection and their subsequent increase during the next three days, suggest the appropriate activation of both Th1/IFN-γ and Th17/IL-17 axes to control intracellular and extracellular parasites during the first four days of infection. After successfully controlling and clearing the parasitic infection, the Th2/IL-4 and Treg/TGF-β axis responses become dominant from day 4 to day 7, limiting inflammatory reactions and promoting tissue repair processes.

Supporting the results of the present study, research previously conducted by Chen et al. demonstrated that the Plasmodium parasite disrupts dendritic cell maturation and antigen delivery during the early stages of infection, leading to impaired activation of the Th1/IFN-γ axis. Consequently, the intracellular antiparasitic immune response weakens, allowing the parasite to persist and continuously stimulate Th1 responses. This eventually causes a shift from an effective Th1 response to an ineffective inflammatory Th17 response against intracellular parasites. While the Th17/IL-17/neutrophil axis effectively controls the parasite during its extracellular phase, it is inadequate for clearing intracellular parasites. As a result, prolonged stability of the Th17/IL-17/neutrophil axis response leads to tissue damage due to inflammation and subsequent complications (18). 

The role of the Th17/IL-17 axis in pathogenesis was highlighted by Bettelli et al., who studied the reciprocal effects of Th17 and Treg cells in malaria. Both were found to be associated with the development of chronic inflammatory tissue complications. Contrary to the Th17 axis, the Treg/TGF-β axis regulates inflammatory responses, particularly the Th17/IL-17 axis, to prevent tissue-related inflammatory complications. Therefore, establishing a balance between the Treg/TGF-β and Th17/IL-17 axes not only controls inflammation but also promotes tissue healing. Conversely, an uncontrolled imbalance favoring the Th17 axis results in tissue destruction, while a deviation toward the Treg axis leads to immune response inhibition and an increased parasite load (19, 20). 

Phytochemical analysis of the extract revealed the presence of Eudesmane sesquiterpene lactones (SLs), including: (1) persianolide A, (2) 4-epi-persianolide A, (3) 3α,4-epoxypersianolide A, (4)11-epi-artapshin, (5)1β,8α-dihydroxy-11α,13-dihydrobalchanin, and (6)1β-hydroxy-11-epi-colartin, as well as (7) methoxylated flavones (1, 2). These compounds are responsible for the observed effects. They could potentially be isolated in large quantities and evaluated in their pure state as new lead candidates for antimalarial treatment alongside standard drugs.

### 5.1. Conclusions

The present study demonstrated an effective synergy between chloroquine and the semipolar extract of *Artemisia kopetdaghensis*. The results showed that, in addition to directly affecting the parasite, the extract can positively influence the health outcomes of the infection by enhancing the host immune system. During the study, the SPE did not cause any severe acute toxicity in the mice; however, minor reversible toxicity was observed at the highest concentration (150 mg/kg). It can be concluded that SPE, when combined with chloroquine, significantly reduces parasitemia, enhances the inhibitory effect on the growth and reproduction of parasites, prolongs the survival time of the infected mice, and helps balance the host immune system.

## Data Availability

The dataset presented in the study is available on request from the corresponding author during submission or after publication.
